# Supplementary material for: Basket to Purkinje Cell Inhibitory Ephaptic Coupling Is Abolished in Episodic Ataxia Type 1
Source: Cells. 2023 May 13;12(10):1382. doi: 10.3390/cells12101382 (PMC10216961; doi:10.3390/cells12101382)
Supplement: Supplementary file 1 [file cells-12-01382-s001.zip › cells-2316064-supplementary.pdf]

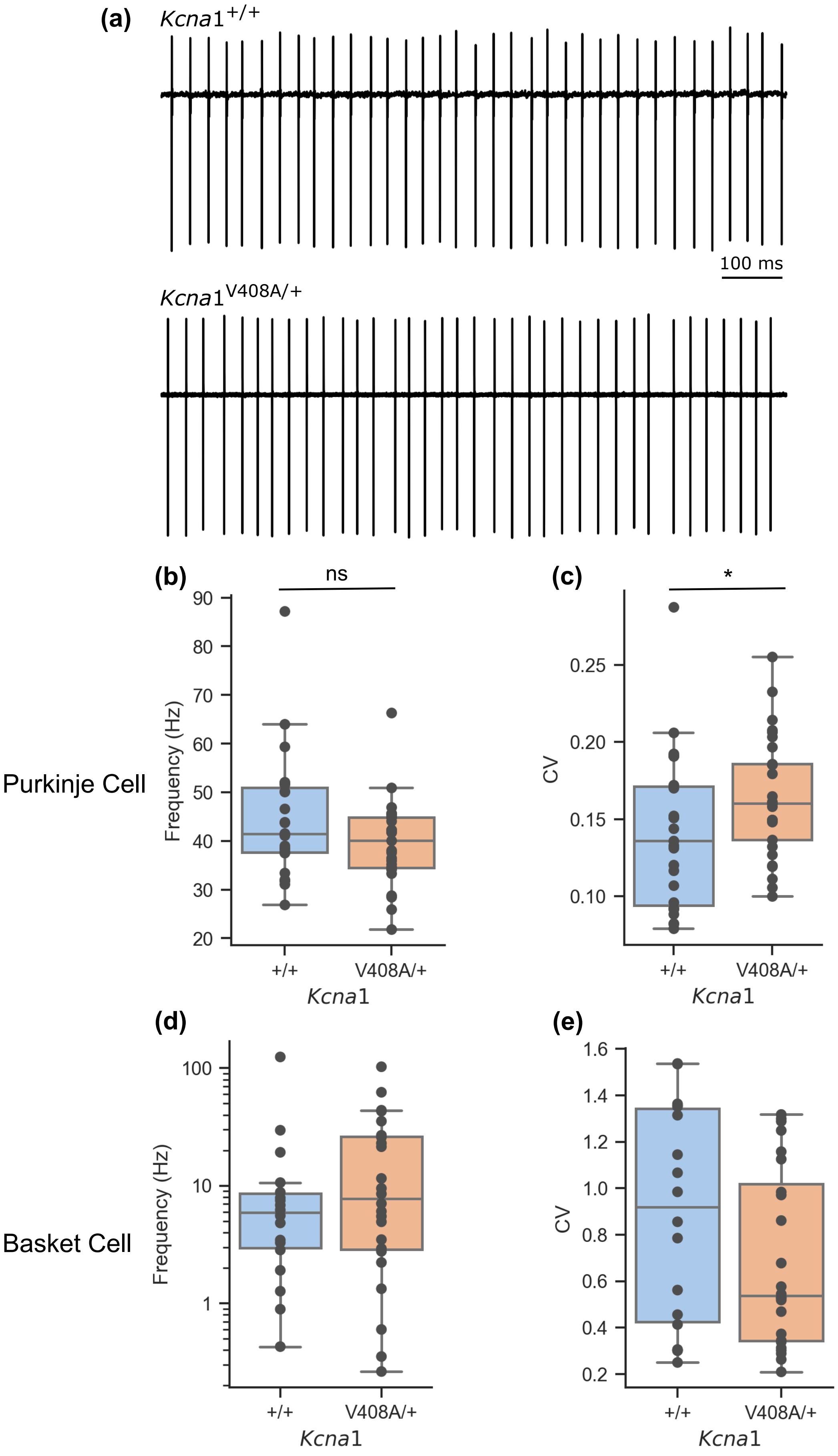

Figure S1. The intrinsic firing of Purkinje cells, but not basket cells is changed in *Kcna1*$^{V408A/+}$ cerebellum. (**a**) representative trace of a firing Purkinje cell recorded from a wild type and *Kcna1*$^{V408A/+}$ cerebellum. (**b**) Average Purkinje cell spontaneous firing frequency. (**c**) The inter-event interval coefficient of variation from the same set of Purkinje cells. Box plot shows interquartile range (IQR) with whiskers at 1.5 IQR (wild type n = 29, *Kcna1*^{V408A} n = 27, *p = 0.046). (**d**) Average basket cell spontaneous firing frequency. (**e**) The basket cell inter-event interval coefficient of variation (wild type n = 18, *Kcna1*$^{V408A/+}$ n = 24).

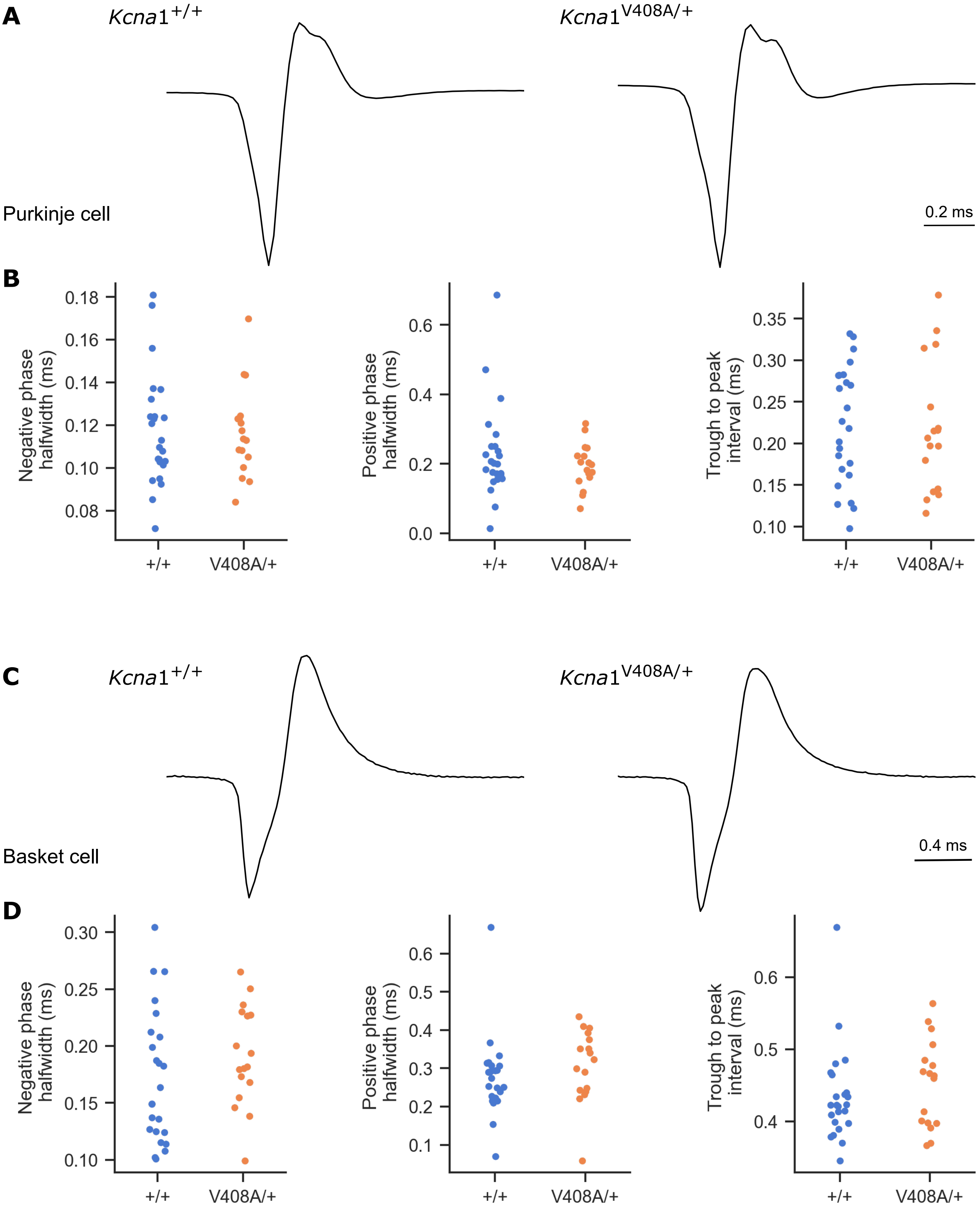

Figure S2.

The profile of Purkinje and basket cell action potentials recorded in cell attached mode are not changed in the *Kcna1*$^{V408A/+}$ cerebellum. *A.* Example current traces from Purkinje cells recorded in cell attached mode, voltage clamped. Traces are average of 100 individual action potentials, scaled in the y-axis to allow direct comparison of shape between genotypes. *B.* Quantification of inidicated phases of Purkinje cell action potential from individual experiments (wild type n = 25, *Kcna1*$^{V408A/+}$ n = 16). *C.* Example current traces from basket cell action potentials recorded in cell attached mode, voltage clamped. Traces are the average of 100 individual action potentials, scaled in the y-axis to allow comparison of shape between genotypes. *D.* Quantification of indicated phases of basket cell action potential from individual experiments (wild type n = 25, *Kcna1*$^{V408A/+}$ n = 16).
